# Rasch analysis of the Brain Injury Screening Tool (BIST) in mild traumatic brain injury

**DOI:** 10.1186/s12883-021-02410-6

**Published:** 2021-09-29

**Authors:** Nusratnaaz Shaikh, Alice Theadom, Richard Siegert, Natalie Hardaker, Doug King, Patria Hume

**Affiliations:** 1 TBI Network, Auckland University of Technology, Auckland, New Zealand; 2 School of Clinical Sciences, Auckland University of Technology, Auckland, New Zealand; 3 Accident Compensation Corporation, Wellington, New Zealand; 4 Sports Performance Research Institute New Zealand, Auckland University of Technology, Auckland, New Zealand; 5 School of Science and Technology, University of New England, Armidale, NSW Australia

**Keywords:** Brain injury, Concussion, TBI, Screening, Assessment, Measurement, Rasch analysis

## Abstract

**Objective:**

To evaluate the psychometric properties of the Brain Injury Screening Tool (BIST) symptom scale in a sample of people with a mild Traumatic Brain Injury (mTBI) through Rasch analysis, and to obtain an interval level measurement score for potential clinical use.

**Materials and methods:**

Data were obtained from 114 adults aged over 16 years, who had experienced at least one mTBI in the past 10 years. Participants were recruited via social media, concussion clinics and sports organisations over a 4-month period between May and September 2020. Participants were asked to compete the symptom scale of the BIST tool via an anonymous online questionnaire. Internal construct validity, dimensionality, person separation index, and differential item functioning of the BIST were examined with Rasch analysis.

**Results:**

BIST in its original form produced a satisfactory item-trait interaction, and good reliability, but was found to be multi-dimensional. Rasch analysis of the full scale with three domains as subtests resulted in acceptable model fit (*χ*^2^(6) =3.8, *p* >  0.05), with good reliability (Person Separation Index = 0.84), and uni-dimensionality. Differential Item Functioning (DIF) analysis displayed no significant DIF effects for sex or age revealing that people responded consistently and similarly to the individual BIST items based on severity of symptom burden.

**Conclusions:**

The 15-item symptom scale of the BIST tool is a psychometrically sound measure of symptom burden following mTBI. The findings provide support for use of both total and sub scale scores for clinical use. Ordinal to interval score conversions are recommended for use when using the scores for research purposes in mTBI.

## Introduction

Mild traumatic brain injuries (mTBIs) are a growing global problem [1]. The impact of increasing prevalence of mTBI is particularly problematic when considering the increasing evidence base that up to half of those affected by an mTBI can experience longer-term effects [2, 3]. These long-term effects include persistent concussion symptoms, impaired cognition, poorer mental health and a decreased ability to function well in everyday life [2, 3]. There is also evidence of an increased risk and earlier onset of longer term health challenges such as stroke and dementia [4, 5]. Evidence shows that early recognition and intervention improves outcomes following mTBI. It is therefore critical to identify those who are at risk of experiencing ongoing problems in order to prevent escalating treatment costs, and higher individual and societal burden.

How TBIs are identified and treated worldwide can vary widely across and within different countries even for moderate and severe TBI injuries [6]. In the case of mTBIs there are several unique challenges in trying to ensure consistent best practice in health care pathways. Firstly, patients present for first medical contact across a range of different services (e.g. school and prison health care teams, sports physicians, physiotherapists, accident clinics, hospital emergency departments). Secondly, medical management is dependent upon a wide range of clinical risk factors (such as prior TBIs or the use of anticoagulants) that professionals are required to be aware of. This is particularly challenging for newly qualified practitioners and those who do not regularly see patients presenting with mTBIs. Thirdly, how international guidelines have been interpreted and implemented varies considerably across contexts (e.g. there are wide differences in assessment processes between sport and non-sport related mild TBI). A further challenge is the differences in health care systems across the globe.

Assessments focusing on loss of consciousness and/or alterations in mental state and associated injury mechanisms have not been found to adequately predict how a person will recover [7]. Further, prognostic models for moderate and severe injuries do not translate well to mTBI [8]. Poor prognosis is based on perceived outcomes on standardised assessment tools such as satisfaction with life, cognitive and neurological functioning as well as symptom experience [9]. The best predictors of poor prognosis following mTBI include, a history of previous TBI, female sex, pre-existing mental health difficulties, delays in seeking medical attention after injury, older age, use of poor coping strategies, and an increased severity of initial symptoms [8, 10]. Within the sports context the Sports Concussion Assessment tool (SCAT-5) [11] includes physical assessment tests, a series of memory questions, such as “which half is it now” and a 22-item symptom scale. However, the authors have acknowledged its limited role in tracking recovery and assisting in return to play/sport decision and the SCAT-5’s use is restricted to those who have been trained in the use of the tool [11]. Additionally, there is currently no evidence to support its applicability to non-sport related mTBIs such as vehicle accidents, assaults and everyday slips, trips and falls. These additional causes together account for 80% of mTBIs [12]. In the research context, the most commonly used tool for assessing impact of mTBI is the Rivermead Post-concussion symptoms Questionnaire (RPQ) [13]. However, the underlying factor structure of the RPQ has been found to vary considerably between samples and over time making it difficult to use either total or subscale scores confidently in outcome prediction. Neither of these tools have been designed to directly inform clinical pathway decision making [14, 15].

To support a more consistent health care management pathway for mTBI and to support the implementation of clinical guidelines for mTBIs, the Brain Injury Screening Tool (BIST) tool was developed by a multidisciplinary working group [16] in order to support the health care decision making process at the first medical contact after injury. The BIST was designed to be brief, able to be completed by any health professional at the first point of medical contact, without the need for specific training. The BIST is designed to support the clinical interview for the mTBI through assessing the level of risk of acute and persistent problems post-injury as well as assessing information about how the injury was sustained, loss of consciousness, and presence of possible risk factors. The BIST also comprises of a symptom scale that asks about possible symptoms in comparison to before the injury. As part of measure development, it is important to explore performance at both a clinical and a measurement level. The BIST tool has previously been found to have good readability (estimated reading age of 6-8 years), ease of completion, good scale reliability, concurrent validity and a three factor underlying structure, with support for use of a total scale score [16]. Rasch analysis builds estimates of true intervals of item difficulty and person ability and transforms ordinal scales into interval measures that may be used in parametric statistical analyses and clinical decision making. For example, calculating individual change scores requires subtraction and this is only legitimate with a unidimensional interval scale such as Rasch provides. The aim of the present analysis was to extend our preliminary evaluation of the psychometric properties of the Brain Injury Screening Tool (BIST) symptom scale using the Rasch analysis, and to obtain a reliable, unidimensional, interval level measurement score for potential clinical use.

## Methods

### Sample

Data were obtained from 114 adults aged 16 to 72 years (32.4 ± 13.6 years), who had experienced at least one mTBI in the past 10 years. Participants were recruited via social media, concussion clinics and sporting organisations between May and September 2020. Ethical approval was obtained from the Auckland University of Technology Ethics Committee (reference: 20/121). The study was conducted in accordance with the study protocol, the Declaration of Helsinki and applicable regulatory requirements. Participants were asked to read the study information sheet and provide an informed consent through an online consent form. The demographic characteristics of the study population are presented in Table 1.

**Table 1:** Socio-demographics and injury-related characteristics for the 114 participants in the study

Participant Characteristics	N (%)
Demographics	
Female	90 (78.9)
European ethnicity	100 (87.7)
In a romantic relationship	75 (65.8)
University/tertiary education	78 (68.4)
Prior Brain Injury	
0	42 (36.8)
1–2	39 (34.2)
≥3	32 (28.1)
Unknown/missing	1 (0.9)
Cause of injury for last TBI	
Accidentally hit by object, person or animal	49 (43.0)
Intentional injury (assault)	10 (8.8)
Fall	37 (32.4)
Vehicle accident	14 (12.3)
Other/unknown	2 (1.8)
Missing	2 (1.8)
Context of injury for last TBI	
Everyday activity	17 (14.9)
Travelling	14 (12.3)
Sport	68 (59.6)
Work/vocational	5 (4.4)
Other	9 (7.9)

### Data collection

Participants interested in taking part in the research were able to access a website through a weblink that provided information about the study and asked for their consent to take part in the research. Respondents were then asked to complete a series of online questions on sociodemographic characteristics and their brain injury history. Respondents were then randomised to receive either the BIST alone or either the symptom scale of the SCAT 5, or the BIST tool and the RPQ to determine concurrent validity*.* Only completed BIST tool data were extracted for this analysis. A sample size of at least 100 participants is required to provide 95% confidence that the item calibrations are within ±0.5 logits [17].

### BIST measure

The BIST was initially designed for those aged 8 years and older and to have a clinical conceptual framework of five subscales: physical, vestibular, cognitive, emotional and sleep. The process of tool development is described elsewhere [16]. The BIST consists of two components. The first component comprises of eight questions used to determine if a patient is at ‘high risk’. This is via a description of what occurred and specific questions aiming to identify any ‘red flags’, or clinical indicators, suggesting that the person may need an urgent referral to hospital e.g., repeated vomiting, post-injury seizure, duration of loss of consciousness. The second component comprises a 15-item symptom report scale. If the injury has occurred within 24 h, the first 11 items are scored as many symptoms cannot be observed until at least 24 h after an injury e.g. sleep quality. People are asked to rate how much they now experience the symptoms listed on a 4-point ordinal scale: 0 (not at all); 1 (mild); 2 (moderate); and 3 (severe). A higher score indicates increased risk of poor recovery and a need for early specialist intervention. The BIST has been found to have good scale reliability and concurrent validity with other symptom measures such as the SCAT-5 and RPQ [16]. Factor analysis previously provided support for use of a total score and three component scores (Physical-Emotional, Cognitive and Vestibular-Ocular) [16].

### Rasch analysis

Rasch analysis provides a robust measurement paradigm for evaluation of person reported outcome measures. The Rasch model proposes that the probability of a person endorsing to a particular item or item response is influenced by the person’s ability (in this case level of symptom severity reported by a person) and the level of difficulty of item (in this case level of symptom severity expressed by the item). For this study, the Windows based statistical package RUMM2030 [18] was utilised to determine fit of the data to the Rasch model. Prior to the main analysis, the suitability of the Rasch model for the analysis was determined by the likelihood-ratio test indicating that the assumptions of the Rating Scale Model were not met (*p* = 0.0001). Hence, the unrestricted Partial-Credit model was applied to conduct the Rasch analysis [19]. The Rasch model requires the scale data to undergo a vigorous iterative process to ensure that the observed pattern of responses meet the model expectations. Analytical criteria to complete the Rasch analysis include item and person fit residuals, item-trait interaction, local independence of items, Differential Item Functioning (DIF) across groups, and unidimensionality. The fit statistics for these criteria are discussed below:

In the case of an acceptable fit to the Rasch model, the overall item and person fit residuals, are expected to have a mean close to zero (SD 1). Individual items should have fit residuals between − 2.50 and + 2.50. An interaction between the item and latent trait reflected by an overall and individual item chi-square fit statistic determines invariance to the scale. A significant item-trait interaction (*p* < 0.05, Bonferroni adjusted) was considered indicative of misfit to the model. The Person Separation Index (PSI) was used as an estimate of reliability, which reflects the ability of a measure to discriminate between persons at different trait levels. A cut-off value above 0.7 for group comparison and 0.8 for individual application was considered acceptable for this analysis. The PSI values can be interpreted similar to Cronbach’s alpha used in classical test theory [20].

A mean residual correlation value of 0.2 was used as an indicator for local response dependency among the items. If local dependency is found between items, they can be combined into a subtest, and the overall fit to the model is re-tested [21]. The Rasch analysis of the BIST was completed in three main analytical pathways: Pathway 1) all 15 items were fitted to the Rasch model without any adjustment; Pathway 2) items from cognitive domains [16] were combined to form a subtest to resolve local dependency issue; Pathway 3) Locally dependent items were combined into three subtests based on the 3-factor structure as presented in the previous work by the authors [16, 21].

Subsequently, we explored DIF across personal factors including age (groups) and gender (groups) using analysis of variance (Bonferroni adjusted). Principal Component Analysis (PCA) was used to examine unidimensionality of the measure, where two groups of items with highest positive and negative loadings on the first principal component of the residuals were derived and compared with independent t-tests [22]. If the percentage of significant *t* tests computed for the lower bound of the binominal confidence interval was below 5%, the scale was accepted to be unidimensional, hence fit to the Rasch model was achieved [23].

## Results

### Sample socio-demographics and injury-related characteristics

The socio-demographics and injury-related characteristics of the 114 participants included in the analysis are provided in Table 1. Participants’ age ranged between 16 and 72, with a mean age of 32.4 years (± 13.6). On average, participants experienced their most recent brain injury on average just over 2 years (mean 2.1 ± 2.3 years) prior to participating in the study. There was a high proportion of European, tertiary educated and female participants within the study. There was good diversity in mechanism and context of injury and history of brain injury.

### Rasch model fit of the BIST

Pathway 1: Initial Rasch analysis was completed using the full (> 24 h) 15-item BIST scale. The fit statistics for individual BIST items with response frequencies for all four categories are reported (in order of easier to the most difficult items) in Table 2. In this analysis, the BIST produced a satisfactory fit (*χ*^2^(30) =21.67, *p* >  0.05) to the Rasch model with good reliability (PSI = 0.91) (see Table 3). However, the binomial test result with greater than 5% significant t-tests indicated that the assumption of unidimensionality for the BIST was not met. The residual correlation matrix identified local dependencies between the items that were represented by the cognitive domain on the underlying factor/conceptual structure of the BIST. Consequently, the BIST items (8, 9, 10, & 11) of the cognitive domain were grouped into a super-item and the analysis was re-run.

**Table 2:** The BIST item fit statistics and frequency distribution of responses categories ordered by Item Difficulty

Item Number	Item description	Item Difficulty^**a**^ (Logits)	Item fit residual	χ2	p	Frequency distribution of response categories (0–3)			
						Cat-0	Cat-1	Cat-2	Cat-3
14	I feel tired during the day	−0.86	− 0.94	2.21	0.33	18	34	34	18
1	Headache (my head hurts)	−0.76	0.71	1.07	0.59	11	53	29	11
15	I sleep a lot more or can’t fall asleep	− 0.54	1.51	0.99	0.61	27	27	34	16
11	I have trouble concentrating	− 0.37	−0.44	1.83	0.40	24	39	**3**	11
8	It takes me longer to think	−0.35	−1.31	1.39	0.50	25	40	27	12
9	I forget things	−0.31	−0.10	0.20	0.90	24	37	33	10
4	I don’t like loud noises	−0.34	0.70	0.25	0.88	25	36	30	11
12	I get easily annoyed	−0.21	−0.01	0.26	0.88	26	38	31	**9**
2	My neck hurts	−0.07	1.23	2.24	0.33	34	42	17	11
3	I don’t like bright lights	0.06	−0.52	0.42	0.81	33	36	27	**8**
13	I feel restless	0.53	0.39	0.41	0.81	46	38	14	**6**
10	I get confused easily	0.54	−0.76	1.46	0.48	48	30	20	**6**
7	I have trouble with my eyesight (vision)	0.68	1.57	3.63	0.16	47	35	18	**4**
5	If I close my eyes, I feel like I am at sea	0.98	−0.55	4.77	0.09	56	30	14	**4**
6	I feel like I will be sick	1.00	−0.04	0.54	0.77	61	30	**9**	**4**

χ2 Chi-square, p probability.

χ2 degree of freedom for items d*f* = 2; Frequency distribution - extreme excluded; Response options in bold were endorsed by less than 10 people.

a = Item Difficulty (location) in logits ordered from easiest (Item 14, Tired) to most difficult (Item 6, Sick) items; negative logit values = easier items,

positive logit values = more difficult items.

**Table 3:** Rasch model summary statistics of the BIST (overall fit of the scale)

Analyses	Item fit residual		Person fit residual		Item-trait Interaction			PSI^**a**^	Cronbach’s alpha	Unidimensional (sig. t-test % p < 0.05) (95% CI)	Local dependency
	Value	SD	Value	SD	χ2	***df***	p		α		
**Pathway 1: All 15 items**	0.10	0.89	−0.18	1.13	21.67	30	0.87	0.91	0.93	No (> 7)	Yes
**Pathway 2: Combined Cognitive symptoms subtest** *(items 8,9,10,11)*	0.26	0.86	−0.11	1.00	26.84	24	0.31	0.90	0.86	No (> 7)	Yes
**Pathway 3: Three Subtests**	0.04	1.13	−0.38	0.97	3.67	6	0.72	0.84	0.77	Yes (4.55)	Yes
**Subtest 1: Physical-Emotional symptoms** (*Items 1,2,3,4,12,13,14,15)*	0.12	0.97	−0.15	0.88	7.20	16	0.97	0.85	0.88	Yes (4.55)	No
**Subtest 2: Vestibular-Ocular symptoms** (*Items 5,6,7)*	0.51	0.40	−0.21	0.88	5.46	6	0.49	0.41	0.57	Yes (0.00)	No
**Subtest 3: Cognitive symptoms** (*Items 8,9,10,11)*	0.19	0.23	−0.33	0.91	1.86	8	0.99	0.83	0.85	Yes (0.91)	Yes
**Ideal values**	0.00	1.0	0.00	1.0			> 0.05^b^	> 0.7		< 5	

*SD* Standard deviation, *χ2* Chi-square, *df* Degree of freedom, *p* Probability, *PSI* Person Separation Index, *CI* Confidence Interval

^a^ PSI value without extremes; ^b^ Bonferroni adjusted

Pathway 2: The second analysis also revealed the BIST produced a satisfactory fit to the Rasch model with a marginal reduction (PSI = 0.90) in reliability of the scale (see Table II). However, the scale showed multidimensionality and the patterns of local dependency emerged for the items of Physical-Emotional, and Vestibular-Ocular domains of the scale. As the BIST has been previously identified to be best represented by a three-factor structure [16] therefore, a subsequent analysis, where the items were grouped into three domain-based subtests (Physical-Emotional, Cognitive, & Vestibular-Ocular), was carried out.

Pathway 3: The final analysis involving three subtests showed improvement in the model fit statistics (*χ*^2^(6) =3.67, *p* > 0.05) and acceptable level of reliability (PSI = 0.84). DIF was examined for age, sex and recovery variables. The analysis displayed no significant DIF effects for these group variables confirming that persons with the same severity (of symptoms) from different age and sex groups and recovery levels respond consistently and similarly to a BIST item. This analysis yielded a strictly unidimensional scale with only 4.55% of the t-tests significant.

Initial testing of the 8-item Physical-Emotional subscale, 3-item Vestibular-Ocular subscale, and 4-item Cognitive subscale were conducted to evaluate the fit of these subscales to the Rasch model (see Table III). The analysis revealed good fit to model and evidence for unidimensionality for all three subscales. However, the PSI value (0.41) for the Vestibular-ocular subscale was found to be below the acceptable cut-off for reliable measurement.

**Fig. 1:**
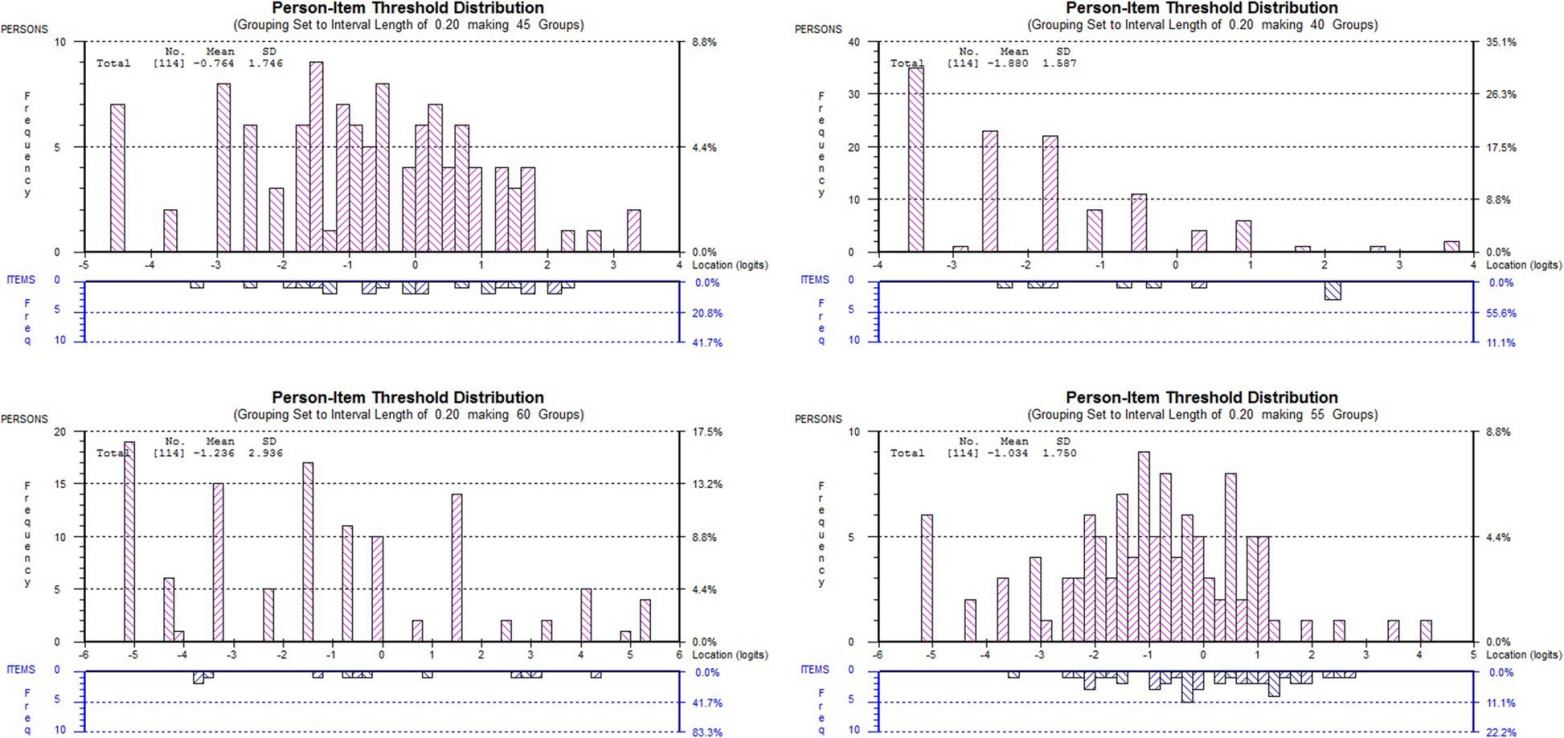
Person Item Threshold Distribution for three subscales and BIST total scale (Pathway 3)

Figure 1 represents the person-item distribution plot of the three BIST subscales and the final solution of the analysis (Pathway Three). Person threshold distribution of the sample appears to be well targeted by the logit continuum for the total scale with over 90% of the sample adequately covered by the total scale. Negligible floor and ceiling effects (< 10%) were found with a small number of persons attaining the minimum and maximum raw Physical-Emotional subscale score. While the observed floor effects for cognitive and Vestibular-Ocular subscale were 16.7, and 31.5% respectively, indicating that these people had a lower level of symptom severity than identified by the scale at the lower ends of the subscales.

**Fig. 2:**
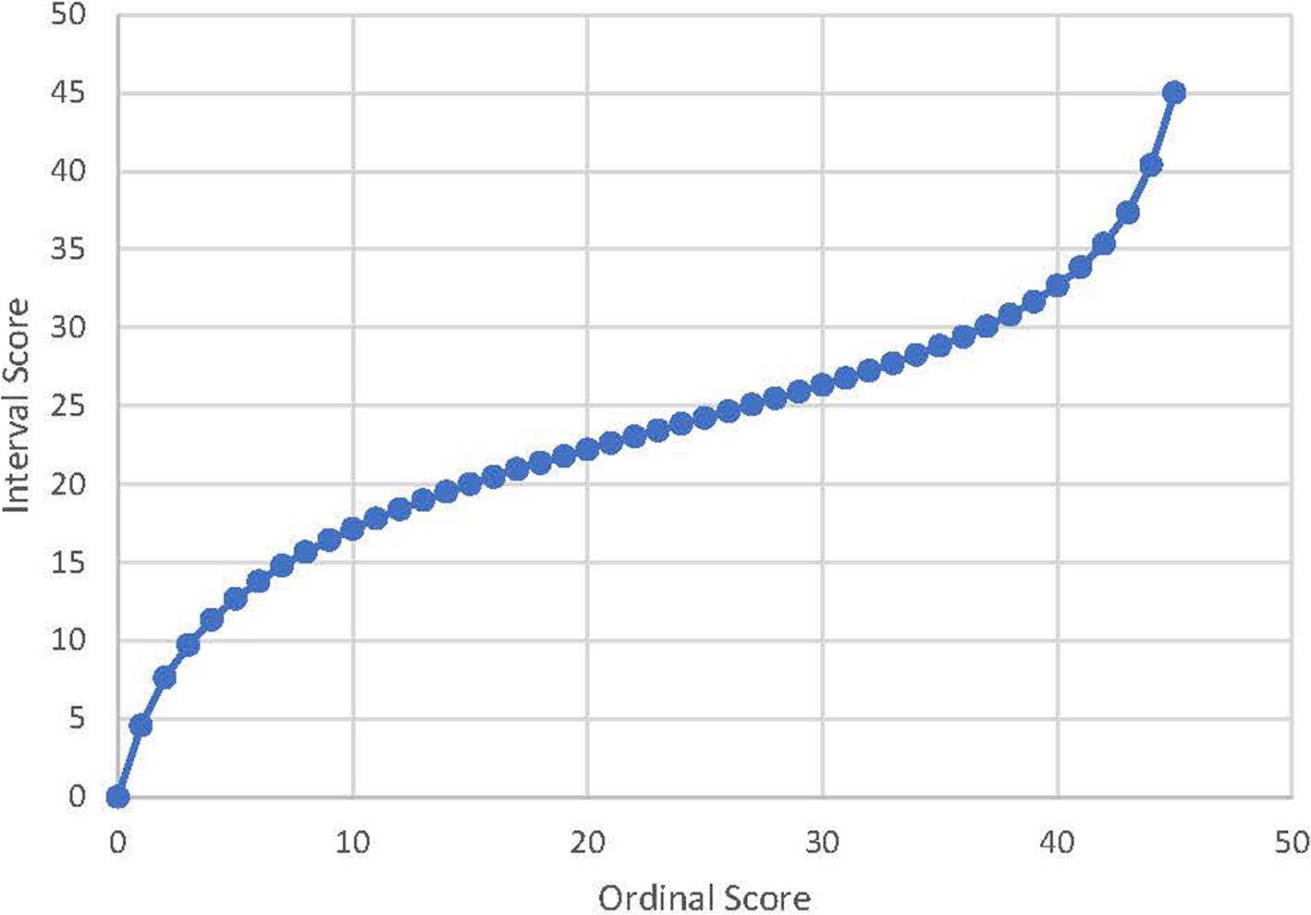
Scatterplot illustrating the relationship between the BIST ordinal summed raw scores and Rasch interval scores

A simple algorithm to convert ordinal BIST scores into the interval-level score is provided in Table 4. The conversion of raw scores for the 15-item BIST does not require any rescoring or modification of the response categories. Absence of DIF indicates that this algorithm is applicable across different age and sex characterises of the sample. However, this can only be used when the respondent data are complete. Figure 2 demonstrates the scatterplot comparing the BIST raw scores with the Rasch transformed interval level score.

**Table 4:** The BIST Conversion Scale - the raw score and corresponding logits and interval score for 15-items

Raw Score	Logits	Interval Score	Raw Score	Logits	Interval Score	Raw Score	Logits	Interval Score
0	−3.208	0.00	16	−0.41	20.47	32	0.513	27.23
1	−2.585	4.56	17	−0.348	20.93	33	0.58	27.72
2	−2.164	7.64	18	−0.289	21.36	34	0.651	28.24
3	−1.879	9.72	19	−0.231	21.78	35	0.727	28.79
4	−1.658	11.34	20	−0.174	22.20	36	0.809	29.39
5	−1.477	12.67	21	−0.118	22.61	37	0.899	30.05
6	−1.323	13.79	22	−0.063	23.01	38	1.001	30.80
7	−1.188	14.78	23	−0.008	23.41	39	1.117	31.65
8	−1.069	15.65	24	0.047	23.82	40	1.254	32.65
9	−0.962	16.43	25	0.103	24.23	41	1.418	33.85
10	−0.865	17.14	26	0.158	24.63	42	1.623	35.35
11	−0.776	17.80	27	0.214	25.04	43	1.896	37.35
12	−0.693	18.40	28	0.271	25.46	44	2.311	40.38
13	−0.616	18.97	29	0.329	25.88	45	2.942	45.00
14	−0.543	19.50	30	0.388	26.31			
15	−0.475	20.00	31	0.449	26.76

## Discussion

This study reports a Rasch analysis of the BIST in adults with a mTBI. The study aimed to reaffirm the psychometric properties of the BIST through evaluation of Rasch model fit statistics and to obtain an interval level measurement score for potential clinical use.

The best fit to the Rasch model was achieved when three groups of locally dependent items of the instrument were combined into three super-items. These super-items corresponded with the three domains of the tool as derived by the preliminary factor analysis: Physical and Emotional domain, Vestibular domain, and Cognitive domain [16]. This can be explained by the shared variance across the items of each domain. The shared variance could be a resultant effect of trait dependence (multidimensionality) or response dependence. Trait dependence within the items of the BIST is plausible as the tool is constructed to measure a unique variable but is comprised of subsets of items that measure somewhat different aspects of that variable. On the other hand, response dependence occurs when a response to an item influences a response to one or more subsequent items (e.g. physical symptom of headache could trigger an emotional response such as restlessness or tiredness). Evidently, it is difficult to distinguish between these two types of violation of local independence [24]. In this case, violation of statistical independence is accommodated by the formation of three super-items as illustrated by Lundgren Nilsson and Tennant [21], and this provided strong evidence for unidimensionality. This process did not require any rescoring or deletion of any of the items, and we were able to derive a conversion table from raw scores to an interval level measure for the total scale.

The relationship between BIST summed raw scores and Rasch transformed interval score (as illustrated in Fig. 2) demonstrates a steep distribution of item thresholds at the upper and lower ends when compared to the considerably flat distribution in the middle. This narrow logit range of the item functioning for the corresponding summed raw scores in the middle indicates a marginal increase in the risk measured by the total BIST scale [See Table 4: 3 unit change in interval score (23 to 26) versus 9 unit change in corresponding raw score (22 to 31)]. Although Rasch transformed interval level scale enhances the precision and robustness of the measure and allows for parametric analysis for the future research, the summed raw scores have a greater responsiveness to detect a clinically meaningful change in respondents. Therefore, we would recommend using the transformed scores for parametric statistical analysis of the subscale or total scale at a group level and using the ordinal level raw score for clinical decision making at an individual level. Further work is required to assess the usefulness of transformed scores in clinical practice. Recent studies have shown that some health professionals lack confidence in assessing and managing TBI [25]. Additionally, the care and advice that patients receive is highly variable. The BIST tool, designed through a collaboration of academics, clinicians and service providers ensures the tool meets the needs of different stakeholders. Its overall aim is to support equitable access to rehabilitation for those at risk of prolonged recovery and to provide a symptom and impact measure that can be used across the spectrum of care to track a patient’s recovery. The tool aims to have applicability across a range of services from primary and secondary care and within other contexts such as school and prison health care teams. On-going consultation with practitioners and clinical research studies to determine clinical utility of BIST scoring are being collected as part of a separate study within clinical settings and determine responsiveness to change.

For the total BIST scale, the PSI of 0.84 was achieved which is marginally short of the cut-off value (≥0.85) for individual clinical use [26]. As discussed earlier, we accounted for dependence by combining the items into subtests and it is believed to have a role in reduction of the PSI for the overall scale [24]. Low reliability for the Vestibular-Ocular subscale can be attributed to a number of reasons including the small number of items in this subscale, items being more difficult hence would be endorsed by a smaller number of people leading to low variance on the latent trait. This supports previous research that shows that higher scores on Vestibular-Ocular subscales indicate increased risk of ongoing problems [27]. Additionally, clinician feedback on the tool has suggested the need for addition of an item – ‘I feel clumsy’ to reflect difficulties with balance and coordination. Addition of this item may enhance the conceptual breadth to improve the reliability of this subscale.

We found relatively larger floor effects for Cognitive and Vestibular-Ocular symptoms subscales which warrants further investigation into sensitivity of these subscales in individuals at the lower (least symptomatic) end of the scale. However, the BIST as a total scale had an appropriate targeting (< 15% floor and ceiling effects) of the clinical population across items and was not found to have an item bias (differential item functioning) across person characteristics such as age and gender. The BIST also met the unidimensionality principle of the Rasch model when domain items were grouped into subtests. With this strong support for the internal validity and reliability of the tool, we recommend application of the BIST total scale and its subscales in their original form in clinical practice in order to measure overall recovery and recovery on the symptoms cluster respectively. Further longitudinal evaluation with a greater sample to determine predictive validity and reproducibility is warranted.

### Limitations

In addition to the limitations discussed above, the authors recognize that the higher representation of females in the study sample may have impacted on the accuracy of the DIF analysis. Additionally, there was a higher representation of people who experienced a mTBI through sport in this sample than shown in epidemiological studies of TBI in the general population. Further research is needed using more representative sampling approaches such as within primary care. Finally, there was no repeated measures data to enable analysis to identify if the person estimates are biased due to response dependence, hence further work to assess if the meaningful change has occurred due to treatment/management or influence of the responses between two occasions.

## Conclusion

The BIST 15 item symptom scale demonstrated good fit to the RASCH model. The findings provide support for use of both the total score and subscales scores for research purposes and ordinal to interval level scores have been provided. Raw scores for the total and subscales should be used for clinical decision making.

## Data Availability

There are ethical restrictions on sharing of de-identified data for this study. The ethics committee has not agreed to the public sharing of data as we do not have the participants’ permission to share their anonymous data. Further, the dataset includes sensitive injury cases and descriptions of the mechanism of injury. It is likely given the nature of the dataset that patients may still be identifiable despite efforts to anonymise the data. Qualified and interested researchers may request access to the data by contacting Professor Kate Diesfield of the Auckland University of Technology Ethics Committee (Phone + 64 9 921 9999 extn: 7837, Email ethics@aut.ac.nz) ethics reference number 20/121.

## Abbreviations

BIST: Brain Injury Screening Tool
mTBI: Mild Traumatic Brain Injury
DIF: Differential Item Functioning
SCAT-5: Sports Concussion Assessment Tool
RPQ: Rivermead Post-concussion Questionnaire
SD: Standard Deviation
PSI: Person Separation Index
PCA: Principal Component Analysis
CI: Confidence Interval
