# Correction to: Rasch analysis of the Brain Injury Screening Tool (BIST) in mild traumatic brain injury

**DOI:** 10.1186/s12883-021-02437-9

**Published:** 2021-11-10

**Authors:** Nusratnaaz Shaikh, Alice Theadom, Richard Siegert, Natalie Hardaker, Doug King, Patria Hume

**Affiliations:** 1 TBI Network, Auckland University of Technology, Auckland, New Zealand; 2 School of Clinical Sciences, Auckland University of Technology, Auckland, New Zealand; 3 Accident Compensation Corporation, Wellington, New Zealand; 4 Sports Performance Research Institute New Zealand, Auckland University of Technology, Auckland, New Zealand; 5 School of Science and Technology, University of New England, Armidale, NSW Australia

**Correction to: BMC Neurol 21, 376 (2021)**

**https://doi.org/10.1186/s12883-021-02410-6**

Following publication of the original article [1], the authors reported an error in Fig. 1 wherein the graph titles “Physical Emotional Symptoms Subscale; Vestibular Ocular Symptoms Subscale; Cognitive Symptom Subscale; BIST Total Scale) were not included in the image. The correct Fig. 1 is shown below.

**Fig. 1:**
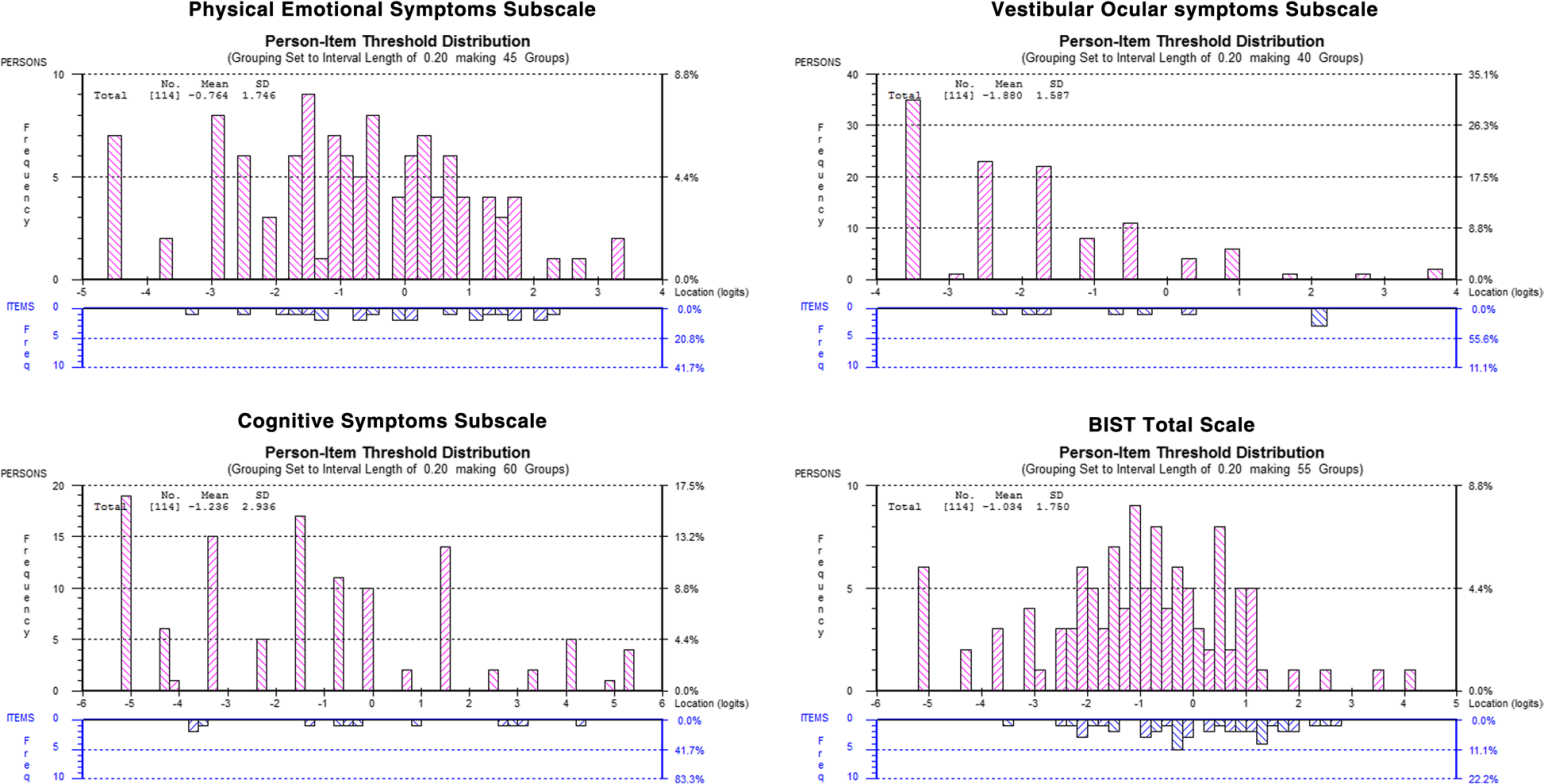
Person Item Threshold Distribution for three subscales and BIST total scale (Pathway 3)

The original article [1] has been updated.
